# Treatment advantage in HBV/HIV coinfection compared to HBV monoinfection in a South African cohort

**DOI:** 10.1016/j.jinf.2020.04.037

**Published:** 2020-07

**Authors:** Tongai G. Maponga, Anna L. McNaughton, Marije van Schalkwyk, Susan Hugo, Chikezie Nwankwo, Jantjie Taljaard, Jolynne Mokaya, David A. Smith, Cloete van Vuuren, Dominique Goedhals, Shiraaz Gabriel, Monique I. Andersson, Wolfgang Preiser, Christo van Rensburg, Philippa C. Matthews

**Affiliations:** a Division of Medical Virology, Stellenbosch University / National Health Laboratory Service Tygerberg, Cape Town, South Africa; b Nuffield Department of Medicine, University of Oxford, Medawar Building, South Parks Road, Oxford, UK; c Division of Infectious Diseases, Department of Medicine, Stellenbosch University / Tygerberg Academic Hospital, Cape Town, South Africa; d Division of Gastroenterology, Department of Medicine, Stellenbosch University / Tygerberg Academic Hospital, Cape Town, South Africa; e Division of Infectious Diseases, Department of Internal Medicine, Faculty of Health Sciences, University of the Free State, Bloemfontein, South Africa; f Division of Virology, Universitas Academic Laboratories, National Health Laboratory Service/University of the Free State, Bloemfontein, South Africa; g Department of Microbiology and Infectious Diseases, Oxford University Hospitals, John Radcliffe Hospital, Headington, Oxford, UK; h NIHR British Research Council, John Radcliffe Hospital, Headington, Oxford, UK

**Keywords:** Hepatitis B virus, HBV, HIV, Treatment, Elimination, Viral load, Tenofovir, Dolutegravir, Hepatocellular carcinoma, South Africa, Coinfection, Sustainable development goals

## Abstract

**Objectives:**

Prompted by international targets for elimination of hepatitis B virus (HBV), we set out to characterise individuals with HBV monoinfection vs. those coinfected with HBV/HIV, to evaluate the impact of therapy and to guide improvements in clinical care.

**Methods:**

We report observational data from a real world cross-sectional cohort of 115 adults with chronic hepatitis B infection (CHB), at a university hospital in Cape Town, South Africa. HIV coinfection was present in 39 (34%) subjects. We recorded cross-sectional demographic, clinical and laboratory data.

**Results:**

Compared to those with HIV coinfection, HBV monoinfected adults were less likely to be HBeAg-positive (p=0.01), less likely to have had assessment with elastography (p<0.0001), and less likely to be on antiviral treatment (p<0.0001); they were more likely to have detectable HBV viraemia (p=0.04), and more likely to have features of liver disease including moderate/severe thrombocytopaenia (p=0.007), elevated bilirubin (p=0.004), and elevated APRI score (p=0.02). Three cases of hepatocellular carcinoma all arose in HBV monoinfection.

**Conclusions:**

Our data demonstrate that individuals with HBV monoinfection may be disadvantaged compared to those with HIV coinfection, highlighting potential systematic inequities in referral, monitoring and treatment.

## Highlights

• We compared HBV monoinfection with HBV/coinfection in a cross-sectional cohort from South Africa.
• HBV/HIV coinfected individuals were more likely to undergo assessment by fibroscan.
• HBV monoinfection was less likely to be treated than HBV/HIV coinfection.
• Indications of severe liver disease were more common in HBV monoinfection.
• Cases of hepatocellular carcinoma all arose in HBV monoinfection.

## Introduction

The burden of chronic infection with hepatitis B virus (HBV) in southern Africa is high, and in this setting the distribution overlaps with populations in which HIV infection is prevalent.^1^ Due to high rates of coinfection, and the overlap in antiviral regimens, there is a pressing need to develop an enhanced understanding of the interplay between these infections, and to capitalise on opportunities for deploying existing HIV infrastructure to improve HBV diagnosis, clinical care and prevention. International Sustainable Development Goals set targets for elimination of HBV as a public health problem by the year 2030.^2^ Developing insights into the characteristics and outcomes of chronic HBV (CHB) monoinfection and HBV/HIV coinfection in real world settings, including lower/middle income countries (LMIC), will underpin improved strategies for monitoring, prognostication, patient stratification and therapy.

A traditional paradigm suggests that individuals with HBV/HIV coinfection might have a worse prognosis from chronic liver disease than those with HBV monoinfection, typically linked with higher rates of HBeAg-positivity and higher viral loads,^1^^,^^3^ lower CD4+ T cell counts, increased incidence of liver fibrosis^4^ and hepatocellular carcinoma (HCC), higher rates of vertical HBV transmission, and higher overall mortality in those with HIV coinfection.^5^ However, this picture is not consistent between populations (even within individual studies)^6^ and the interplay between viruses may be affected by specific characteristics of the host or viral population,^7^ as well as by changes in treatment guidelines over time.

Lamivudine (3TC) and tenofovir (most commonly in the form of tenofovir disoproxil fumarate, TDF) are nucleos(t)ide reverse transcriptase inhibitor (NRTI) agents with established track records of safety and tolerability in both HIV and HBV infection. Long-term 3TC use is hampered by a high rate of selection of resistance,^8^ while TDF is favoured as the genetic barrier to resistance is high.^9^ HIV treatment is now initiated irrespective of clinical or immunological status,^10^ underpinned by ‘90-90-90’ targets, seeking to have 90% of cases diagnosed, 90% of these on treatment, and 90% of these virologically suppressed.^11^

TDF is one of the recommended choices for the NRTI backbone of first line cART for HIV.^12^ In South Africa, second line NRTI combinations also include TDF or 3TC,^13^ and national ART guidelines recommend HBsAg testing for patients switching from a first line regimen to avoid acute hepatitis flares in case of TDF withdrawal. In HBsAg positive individuals, TDF is continued as a component of second line cART. Thus the majority of those with HIV/HBV coinfection receive TDF, either as routine first-line therapy, or incorporated into second-line regimens.

In contrast, for HBV monoinfection, TDF is prescribed only for a subset of patients, based on algorithms that incorporate assessment of the patient (age and sex), virologic status (HBV DNA viral load) and the presence of underlying fibrotic or inflammatory liver disease (ALT, elastography score, ultrasound appearance, biopsy results).^14^, ^15^, ^16^ WHO targets for 2030 aim for 90% of HBV cases to be diagnosed, and for 80% of those eligible for treatment to be receiving it.^17^ However, only a small minority of those living with CHB are currently aware of their status, and laboratory and radiological assessment are not accessible in many settings.^18^ Current assessment using WHO guidelines therefore typically misses a high proportion of patients who should be treated.^19^

Recognising the need for improved characterisation of CHB infection, both alone and in combination with HIV coinfection, we have undertaken a cross-sectional observational analysis of adults with CHB in an urban setting in South Africa. We set out to explore the differences between individuals with monoinfection and coinfection, and to identify inequities that may arise as a combined result of referral bias and differences in care provision. Our approach included specific focus on the influence of discrepancies between HBV and HIV guidelines for antiviral therapy. Our aim is to identify areas in which local and regional clinical practice can be strengthened, with the wider potential to inform future guidelines for care of adults with CHB.

## Methods

### Clinical cohort

We undertook a real world study, representing a cross-sectional observation of adults in clinical care at Tygerberg Hospital, a tertiary referral hospital in Cape Town, South Africa. We recruited adults with CHB infection, with or without HIV coinfection, attending routine clinical follow-up in hepatology and infectious diseases outpatient clinics. Healthcare workers at local HIV and primary care clinics within the referral area are encouraged to send all patients diagnosed with HIV-HBV coinfection to the Tygerberg Division of Infectious Diseases clinic for counselling and baseline investigations including clinical assessment, laboratory tests and elastography.

Surrounding clinical centres also refer adults testing HBsAg-positive for assessment and follow-up in the Division of Gastroenterology. Patients are followed at the clinic at intervals according to the baseline findings, typically attending at intervals of six months. Thus, our cohort represents prevalent chronic disease in this community, rather than new incident infections. Cases were defined as being HBsAg-positive (having been under follow-up for a period of >6 months) and were recruited into a cross-sectional cohort (Oxford-South Africa Hepatitis Cohort, ‘OxSA-Hep’), commencing July 2018. We here present the results of a planned interim analysis of data after 12 months of recruitment.

We recorded treatment with antiviral therapy at the time of recruitment to the study, routine clinical laboratory data (including serological markers of HBV infection, creatinine, liver function tests and platelet count), and documented elastography scores when the patient had been assessed by fibroscan. We recorded data in a LabKey database,^20^ using a unique pseudonymised patient ID number.

### Generation and analysis of laboratory data

Biochemical and serological data were generated on the Cobas 6000 series e601 module analyzer (Roche Diagnostics GmbH, Germany), including alanine aminotransferase (ALT) and aspartate aminotransferase (AST) and serum bilirubin (BR). Platelet counts were obtained using the Advia 2120i analyzer (Siemens Healthcare Diagnostics Inc, USA). HIV and HBV viral loads were measured using Cobas Ampliprep/Taqman tests (Roche Molecular Diagnostics, the Netherlands).

Local guidelines were used to define thresholds.^21^ We defined the upper limit of normal (ULN) for ALT as 19 U/L for females and 30 U/L for males,^16^^,^^14^ ULN for AST as 40 U/L, ULN for BR as 17 mmol/L. We calculated liver fibrosis scores, AST to Platelet Ratio Index (APRI) and Fibrosis-4 (FIB-4) as follows:

• APRI = (AST / ULN AST) / (Platelet count x 100)
• FIB-4 = (Age in years x AST) / (Platelet count * √ALT)

We used thresholds of FIB-4 >3.25 (97% specificity, and positive predictive value of 65% for advanced liver fibrosis^22^), and APRI >2 (91% specific for cirrhosis).^23^^,^^24^

We measured renal function based on estimated glomerular filtration rate (eGFR) as follows:

• 186 x (creatinine (µmol/L)/88.4)^−1.154^ x (age in years)^−0.203^ x (0.742 if female) x (1.210 if black).^25^

Normal renal function is defined as eGFR ≥ 90 ml/min/1.73m^2^. We grouped together individuals with renal impairment, defined as chronic kidney disease (CKD) Stage II (eGFR 60-89 ml/min) or Stage III (eGFR 30-59 ml/min). We defined moderate/severe thrombocytopaenia as a platelet count <75 × 10^3^/µl.^26^

### Elastography and imaging

Elastography to quantify liver stiffness (an assessment of inflammation and/or fibrosis) is not undertaken consistently in this setting, but is performed in a subset of patients at the discretion of the clinical team using the Fibroscan 402 (Echosens, Paris, France). Elastography scores are not reliable in individuals with a high body mass index or during pregnancy, and would not be undertaken in these circumstances. Due to the cross-sectional nature of the cohort, we recorded elastography scores at a single time point only. HCC screening is not undertaken as part of routine practice, as access to imaging and biomarkers for surveillance is limited. Therefore, a diagnosis of HCC is usually made on the basis of an observed deterioration in clinical status or laboratory markers, followed by imaging in selected cases.

### Treatment indications and outcomes

We have referred to HBV treatment recommendations published by the UK National Institute for Clinical Excellence (NICE),^14^ the European Association for the Study of the Liver (EASL),^15^ and South African national guidelines.^16^ All concur in advising tenofovir or entecavir as first-line options, and recommend that stratification for therapy should be based on laboratory parameters, on the presence and extent of existing liver disease assessed by imaging and/or biopsy, and on consideration of other individual clinical and demographic factors.

Due to resource constraints, liver biopsy is rarely undertaken in this setting and we have primarily used laboratory parameters to assess treatment eligibility. South African guidelines suggest consideration of therapy for individuals who are HBeAg-positive with HBV DNA >20,000 IU/ml and ALT above ULN, or who are HBeAg-negative with HBV DNA >2,000 IU/ml and ALT above ULN, with the aim of achieving durable suppression of HBV DNA to low or undetectable levels and normalisation of ALT.^16^ Individuals with HIV coinfection are routinely treated with first line cART that includes HBV-active agents.^13^ In practice in this setting, TDF is therefore first line therapy for CHB, both in the presence and absence of HIV infection.

### Statistical analysis

We analysed data using GraphPad prism v 8.0.1. For continuous variables, we used Mann Whitney Test; for categorical variables we used Fisher's Exact Test. Linear regression was used to assess associations between two continuous variables. For multivariate analysis, we performed multivariate logistic regression using bayesglm function of the R package.

### Ethics

Ethics approval was provided by University of Oxford Tropical Research Ethics Committee (ref. OXTREC 01-18) and Stellenbosch University Human Research Ethics Committee (HREC ref. N17/01/013). All participants provided written valid informed consent. A STROBE statement to support the quality of this observational study is provided (Suppl. Table 1).

## Results

### Representation of HBV monoinfection and HIV/HBV coinfection in an adult cohort

**Fig. 1:**
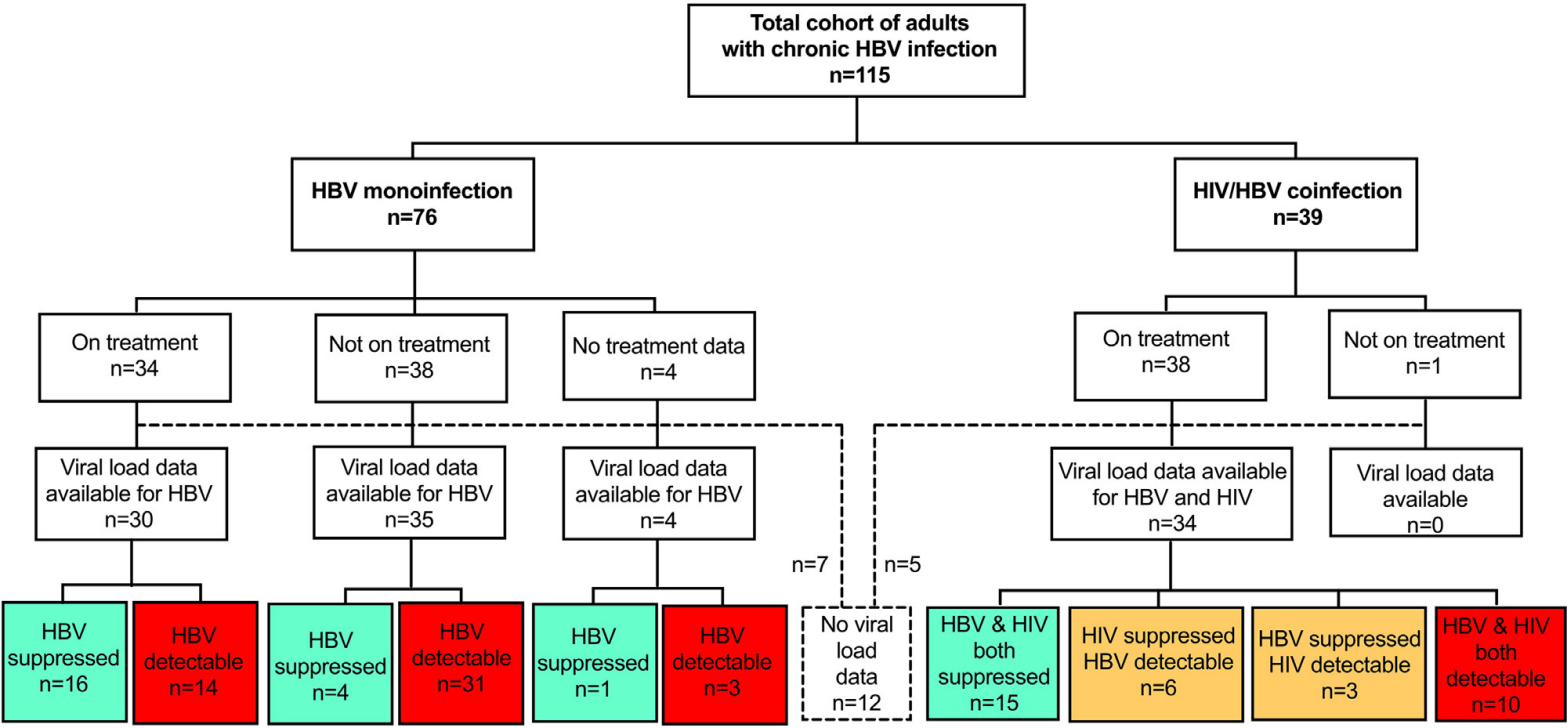
Summary of cohort of 115 adults with HBV infection from Cape Town, South Africa, showing numbers with HBV monoinfection and HIV/HBV coinfection, those receiving therapy and those with suppressed viraemia. Viral load data were not available in 12 cases, of whom 7 were HBV monoinfected and 5 were HBV/HIV coinfected (shown in dashed lines and box). Green boxes indicate number with viraemia suppressed below the limit of detection, yellow indicate number with one virus suppressed and the other detectable, red indicate number with no viraemic suppression.

**Fig. 2:**
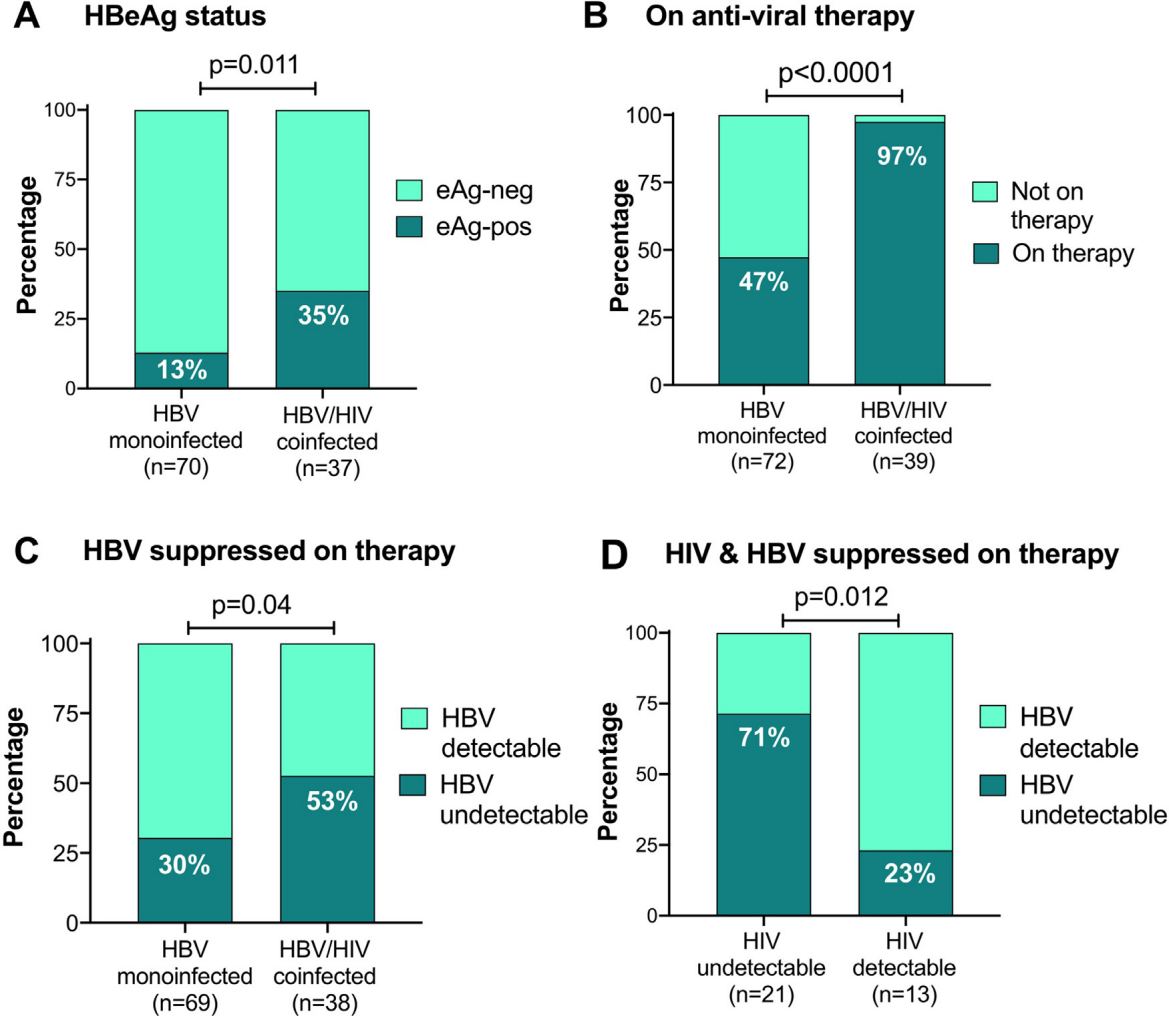
Characteristics of adults with CHB, based on HBeAg status, HBV therapy and virologic response to therapy, comparing those with HBV monoinfection vs HBV/HIV coinfection from a cross-sectional study in Cape Town, South Africa. A: Proportion of each group testing HBeAg-positive. B: Proportion of monoinfected vs coinfected adults receiving antiviral therapy; C: Proportion of monoinfected vs coinfected adults with HBV DNA viral load suppressed below the limits of quantification (<20 IU/ml) on antiviral therapy. D: HBV/HIV co-infected individuals only, showing proportion who have suppressed HIV and/or HBV viral load below the limit of quantification on antiviral therapy. In all cases, p-values calculated by Fisher's Exact Test, and the number of individuals represented in each group is shown in brackets below the columns.

We recruited 115 adults with HBV infection (57% male), representing 76 adults with HBV monoinfection (66%) and 39 with HBV/HIV coinfection (34%) (Fig. 1; Table 1). The full metadata for this cohort are available in Suppl. Table 2. The majority of participants were of South African origin (98/115, 85%; Suppl. Fig. 1). There was no significant difference in sex, age or body weight between the monoinfected and coinfected groups (p=0.24, 0.51, and 0.23 respectively; Table 1). HBeAg-positive status was significantly more common among individuals with HIV coinfection (39%) compared to HBV monoinfected individuals (14%); (p=0.01; Table 1; Fig. 2A).

**Table 1:** Summary of clinical and laboratory parameters recorded from a cohort of 115 adults with HBV infection in Cape Town, South Africa, comparing groups with HBV monoinfection (n=76) versus HIV/HBV coinfection (n=39). For extended version of table, see Suppl data table 3.

	Whole Cohort	HBV monoinfection	HIV/HBV coinfection	p-value^a^
Sex M:F (% male)	65:50 (57%)	46:30 (61%)	19:20 (49%)	0.24
Median age in years at enrollment^b^ (IQR)	44 (36-53)	45 (35-54)	41 (37-50)	0.51
Median body weight in kg^c^ (IQR)	73 (60-84)	75 (60-86)	71 (60-80)	0.23
Proportion HBeAg positive^d^ (%)	22/107 (21%)	9/70 (13%)	13/37 (35%)	0.011
Proportion with elevated ALT above ULN^e^ (%)	52/113 (46%)	36/74 (49%)	16/39 (41%)	0.55
Proportion with elevated AST above ULN^f^ (%)	31/95 (33%)	20/58 (35%)	11/37 (30%)	0.66
Median BR^g^, mmol/L (IQR)	7 (5-14)	9 (6-17)	5 (4-9)	0.004
Proportion with elevated BR > ULN^g^ (%)	19/109 (17%)	16/70 (23%)	3/39 (8%)	0.06
Median platelet count^h^^,^ x10^3^ / µl (IQR)	236 (163-297)	227 (156-294)	240 (170-307)	0.41
Proportion with thrombocytopaenia^h^ (%)	11/106 (10%)	11/67 (16%)	0/39 (0%)	0.007
Proportion with APRI score >2	8/92 (9%)	8/55 (15%)	0/37 (0%)	0.02
Proportion with FIB-4 score >3.25	21/92 (23%)	16/55 (29%)	5/37 (14%)	0.13
Proportion assessed by elastography^i^ (%)	48/115 (42%)	13/76 (17%)	35/39 (90%)	<0.0001*
Median elastography score, kPa (IQR)	6.1 (4.8-9.0)	7.8 (5.4-10.3)	5.8 (4.5-8.2)	0.15
Proportion with elastography score >9kPa (%)	12/48 (25%)	5/13 (38%)	7/35 (20%)	0.26
Proportion with hepatic complications^j^ (%)	20/112 (16%)	15/73 (21%)	5/39 (13%)	0.12
Proportion with CKD stage II or III^k^	16/111 (14%)	9/72 (13%)	7/39 (18%)	0.79

IQR = inter-quartile range; ALT = alanine transferase; ULN = upper limit of normal.

a p-value for categorical variables by Fisher's Exact Test, and for continuous variables by Mann Whitney test.

b Age available for 115 individuals.

c Body weight available for 111/115 individuals.

d HBeAg status available for 107/115 individuals.

e ALT available for 113/115 individuals, with ULN defined as 19 iu/ml for females and 30 iu/ml for males.

f AST available for 95/115 individuals, with ULN defined as 40 iu/ml.

g BR available for 109/115 individuals; ULN defined as 17 mmol/L.

h Platelet count available for 106/115 individuals; moderate/severe thrombocytopaenia defined as platelet count <75 × 10^3^ / ul ^27^.

i Elastography data available for 50/119 individuals.

j Hepatic complications reviewed for 108/112 individuals, defined as a documented diagnosis of HCC and/or cirrhosis.

k CKD (chronic kidney disease) stages II and III defined as eGFR <90 ml/min/1.73m^2^.

⁎ also significant on multivariate analysis.

### Antiviral therapy and virologic outcomes in HBV monoinfection vs HIV/HBV coinfection

Duration of antiviral therapy was recorded for 36 individuals with HBV monoinfection and 37 individuals with HIV coinfection (median 38 vs. 62 months, respectively; p=0.1). As anticipated, based on HIV treatment guidelines,^13^ a significantly greater proportion of the coinfected group was on antiviral therapy compared to the monoinfected group (97% vs. 47% respectively, p<0.0001; Fig. 2B; Table 2). Accordingly, HBV DNA was more likely to be suppressed below the limit of detection in coinfected than monoinfected patients (53% vs 30%, respectively; p=0.04; Fig. 2C; Table 2), and absolute values for HBV DNA viral load (VL) were also significantly lower in the coinfected group than the monoinfected (p=0.04; Suppl Fig. 2A; Table 2).

**Table 2:** Summary of antiviral therapy treatment and outcomes from a cohort of 115 adults with HBV infection in Cape Town, South Africa, comparing features of those with HBV monoinfection (n=76) versus HIV/HBV coinfection (n=39).

	Whole Cohort	HBV monoinfection	HIV/HBV coinfection	p-value^a^
Proportion on antiviral treatment^b^ (%)	72/111 (65%)	34/72 (47%)	38/39 (97%)	<0.0001*
Median duration of therapy^c^, months (IQR)	45 (25-77)	38 (17-69)	62 (29-81)	0.11
Median HBV DNA viral load IU/ml^d^ (range)	42 (0-1.7 × 10^8^)	86 (0-1.7 × 10^8^)	0 (0-1.1 × 10^8^)	**0.04**
Proportion of HBV viraemia undetectable^e^	41/107 (38%)	21/69 (30%)	20/38 (53%)	**0.04**

a p-value for categorical variables by Fisher's Exact Test, and for continuous variables by Mann Whitney test.

b Antiviral treatment data available for 111/115 individuals.

c Duration of therapy defined for 36 HBV monoinfected and 37 HBV/HIV coinfected individuals.

d HBV DNA viral load available for 69/76 HBV monoinfected and 38/39 HIV coinfected individuals.

e Undetectable HBV DNA defined as viral load in serum below limit of detection of assay (typically HBV DNA <20 IU/ml).

⁎ also significant on multivariate analysis.

In the HBV/HIV coinfected group, 38/39 adults were on antiviral therapy that included HBV-active agents (TDF and/or 3TC); one patient was not on therapy. HIV VL data were available for 35/38, being undetectable in 22/35 (63%), and detectable but <1000 RNA copies/ml in a further 5/35 (14%). Overall, suppression of HIV viraemia was associated with suppression of HBV viraemia (p=0.01, Fig. 2D; Suppl Fig. 2B). Among individuals with undetectable HIV VL, HBV DNA was still detectable in 6/21 (29%). Among these, one was being treated with 3TC as the only HBV-active agent, with HBV DNA VL 1.0 × 10^5^ IU/ml. Suppression of HIV viraemia suggests reasonable adherence to cART, and the high HBV viral load may therefore be consistent with 3TC resistance. The other five were receiving TDF-based combination therapy, making drug resistance a less likely explanation for HBV viraemia (HBV VL 20-5444 IU/ml). Treatment start date was ≥15 months prior to the date of recruitment (recorded in all 6 cases), making it unlikely that HBV viraemia was detectable because therapy had only recently been instituted.

### Proportion of untreated HBV monoinfected patients who meet treatment criteria

We reviewed the data for 38 HBV monoinfected individuals off therapy to determine the proportion of these who met treatment criteria. Using South African guidelines,^16^ 8/38 (21%) were treatment eligible, comprising individuals with ALT >ULN of whom four were HBeAg-positive with HBV DNA >20,000 IU/ml, and four were HBeAg-negative with HBV DNA >2,000 IU/ml.

### Prevalence of liver disease: laboratory data

**Fig. 3:**
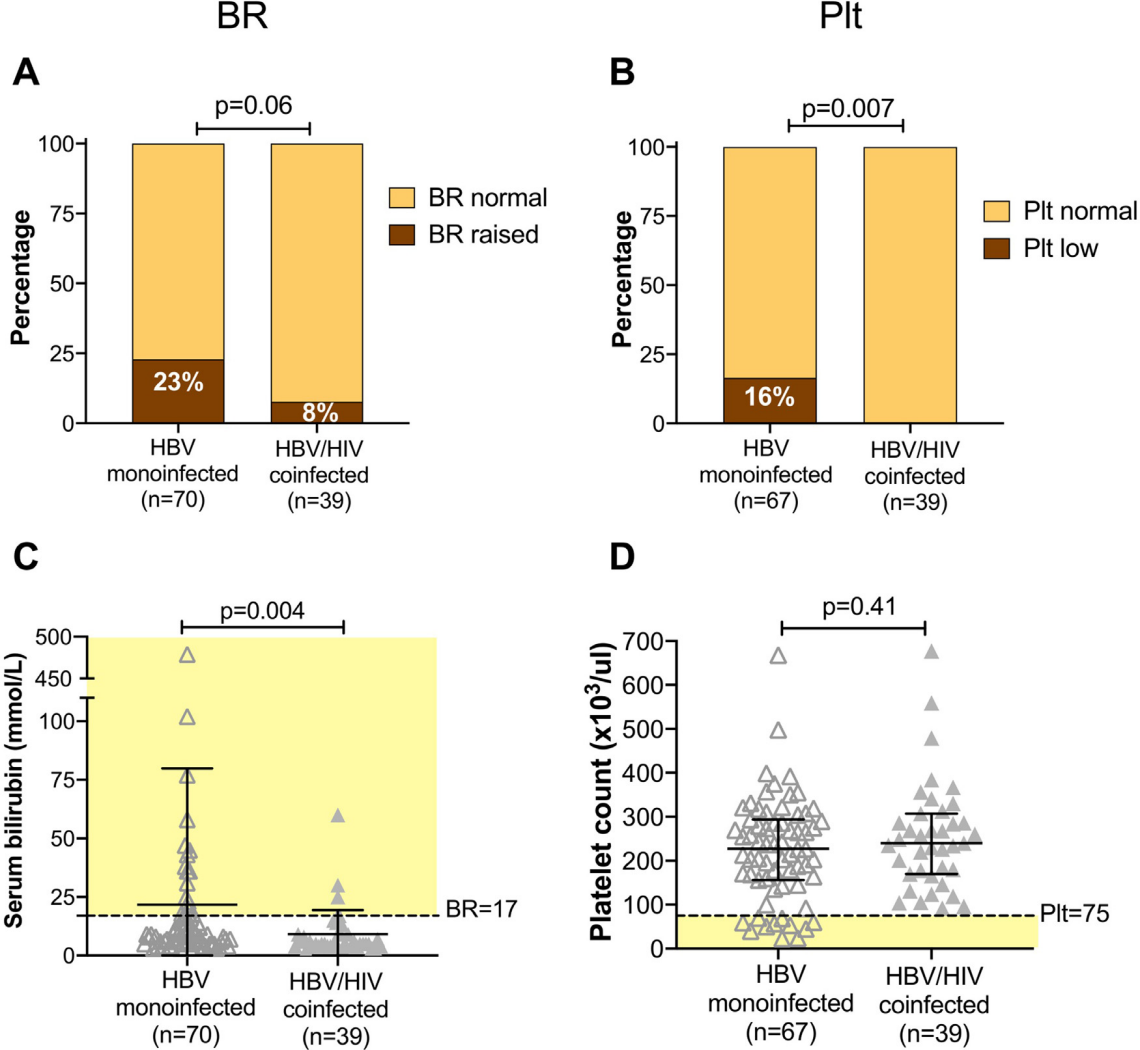
Assessment of liver disease in adults with HBV monoinfection vs HBV/HIV coinfection in a cross-sectional study in Cape Town, South Africa based on serum bilirubin and platelet count. BR - serum bilirubin; Plt - platelet count. Panels A and B: p-values by Fisher's Exact Test comparing the number in each group falling above and below specified thresholds (BR>17 mmol/L; moderate/severe thrombocytopaenia defined as plt <75 × 10^3^/µl). Number in each group indicated in brackets under the columns; this varies according to data availability. Panels C and D: p-values by Mann Whitney test; bars indicate median and IQR. Pale yellow shading shows population defined as following outside reference range. C: serum bilirubin levels are significantly elevated in the group with HBV monoinfection. D: Distribution of plt highlights all of those with moderate/severe thrombocytopaenia (below dashed line) are in HBV monoinfected group.

We assessed laboratory data and elastography scores as markers for underlying liver disease. There was no significant difference in ALT or AST between monoinfected vs. coinfected individuals (p=0.8 and p=0.6, respectively). For BR, the absolute values were significantly higher in those with monoinfection (p=0.004), and a higher proportion of the population had an elevated value in the moninfected group (23%) vs. the coinfected group (8%), although this difference did not reach statistical significance (p=0.06); Fig. 3A,C. Thrombocytopaenia can be an indicator of chronic liver disease as a result of a variety of mechanisms, including splenic sequestration and myelosuppression.^26^ Strikingly, all the individuals with moderate/severe thrombocytopaenia were in the HBV monoinfected group, representing 16% of this group vs. 0% in those with coinfection (p=0.007); Fig. 3B,D, suggesting a subgroup of individuals with established liver disease.

**Fig. 4:**
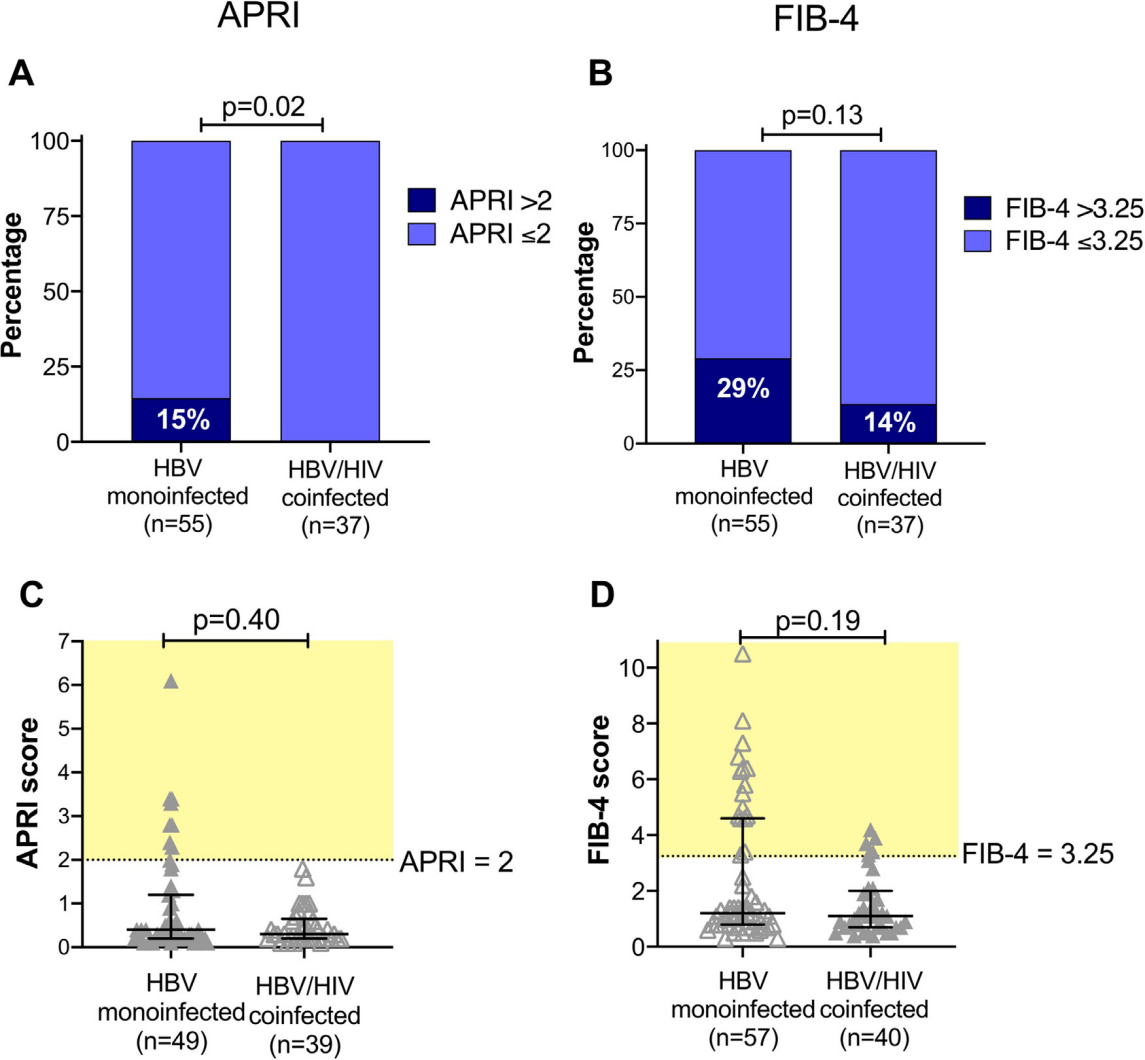
Assessment of liver disease using fibrosis scores derived from laboratory data in adults with HBV monoinfection vs HBV/HIV coinfection in a cross-sectional study of adults in Cape Town, South Africa. APRI - AST to Platelet Ratio Index; FIB-4 - Fibrosis-4 score (for formulae, see methods). Number in each group indicated in brackets under the columns; this varies according to data availability. Panels A and B: p-values by Fisher's Exact Test comparing the number in each group falling above and below the threshold for cirrhosis/fibrosis (APRI >2; FIB-4 >3.25). Panels C and D: p-values by Mann Whitney test and bars indicate median and IQR. Pale yellow shading shows population defined as falling above the defined threshold for liver disease. No significant difference in median value for either score, but the scatter plots show that individuals with scores above the threshold are predominantly in the mono-infected group.

We calculated fibrosis scores from laboratory data, finding a significantly higher proportion of the HBV monoinfected group had APRI scores predictive of cirrhosis (p=0.02; Fig. 4A). There was a trend in the same direction, although non-significant, for FIB-4 (p=0.1, Fig. 4B).

### Prevalence of liver disease: elastography and clinical data

Due to limited clinical availability, elastography is not routinely undertaken, and data were only available in 48/115 patients (42%). Individuals with HBV/HIV coinfection were more likely to have been assessed by elastography, in whom it was undertaken in 35/39 cases (90%) compared to only 13/76 (17%) of individuals with HBV monoinfection (p<0.0001; Table 1). The median elastography score was higher in the HBV monoinfected group compared to those with coinfection (7.8 kPa vs 5.8 kPa), and a greater proportion of the HBV monoinfected group met the stringent treatment threshold of 6 kPa (9/13 (64%) monoinfected vs. 16/35 (47%) coinfected patients).^14^ Neither of these differences reached statistical significance (p=0.2, in both cases), but this comparison is limited by small numbers. There was no difference in ALT values between the monoinfected vs coinfected patients with documented fibroscan scores (median ALT 22 vs. 23 U/L, respectively), suggesting that elastography was not systematically undertaken for HBV monoinfected patients with biochemical evidence of inflammatory liver disease.

We reviewed clinical records for complications reported by specialist doctors in 112/115 cases (data not available for three patients). Among these, 20/112 (18%) had hepatic complications of HBV infection, including 18 cases of cirrhosis (16% prevalence) and 3 cases of HCC (3% prevalence); two patients had both cirrhosis and HCC. The prevalence of hepatic complications was almost twice as high in HBV monoinfection (15/73; 21%) compared to HBV/HIV coinfection (5/39; 13%), although this difference did not reach statistical significance due to small numbers in each group (p=0.1; Table 1). All HCC arose in males (age 30, 53 and 60) and occurred in the context of HBV monoinfection. One patient with both HCC and cirrhosis was on TDF therapy, whereas the other two were untreated at the time of data collection. Due to the small numbers and relatively short duration of follow-up, we cannot draw any conclusions regarding the risk factors or epidemiology of HCC based on this study, but the HCC arising in a 30-year old is consistent with the early age at which this malignancy arises in sub-Saharan African populations.^27^

### Evidence of renal dysfunction

Chronic kidney disease (CKD) can arise in association with HIV infection, and is also a potential side effect of TDF therapy.^28^ Therefore, we examined this cohort for the presence of CKD based on calculation of eGFR, available for 111 individuals. Due to small numbers, we grouped together CKD stage II and III, finding a prevalence of 16/111 (14%). There was no difference in the prevalence of CKD between individuals with HBV monoinfection vs. HBV/HIV coinfection (CKD prevalence 9/72 (13%) vs. 7/39 (18%), respectively, p=0.6), and no evidence of nephrotoxicity among those on TDF (CKD present in 9/65 (14%) of treated individuals, vs. 7/43 (16%) of untreated; p=0.8). While we cannot exclude the possibility that selection bias for treatment might mean TDF is more frequently started in individuals with normal renal function, these results are nevertheless reassuring in suggesting no significant on-treatment nephrotoxicity.

### Multivariate analysis

Based on small numbers, we were underpowered to identify significant associations based on multivariate analysis. However, the relationship between HBV/HIV coinfection and undergoing assessment by elastography and being prescribed antiviral therapy remained significant on multivariate analysis (Tables 1 and 2, footnotes).

## Discussion

### Context and primary conclusions

Developing a detailed understanding of the characteristics of CHB, and the clinical interplay with co-endemic HIV, is important to drive forward improvements in care provision. Although successful HBV vaccination campaigns are well established, modelling studies predict long timeframes to elimination.^29^^,^^30^ This cross-sectional cohort provided an opportunity to make real world comparisons between adults with HBV monoinfection and HBV/HIV coinfection, and to reflect on the systematic inequities and differences in the structure of clinical care provision which might contribute to different disease outcomes.

In this setting, we confirmed a significantly higher prevalence of HBeAg-positive status in those with HIV coinfection, associated with higher HBV viral loads and acceleration of inflammatory or fibrotic liver disease. However, the observational data we have collected indicate that - in this setting - the monoinfected group is disadvantaged compared to those with coinfection. Worse outcomes in HBV monoinfection may stem from the sustained neglect of HBV as a public health problem,^18^ complex algorithms for HBV treatment stratification, inconsistent access to TDF therapy,^31^ and referral bias. It seems likely that the broad patterns we observe are generalisable across southern Africa, given the widespread investment that has been made in HIV infrastructure versus the lack of consistent HBV services. However, our focus on an urban tertiary referral centre is situation-specific, and more data are required to explore different regional patterns.

A previous report also documents elevated rates of severe fibrosis in HBV monoinfected compared to their HBV/HIV coinfected counterparts,^32^ although this observation is not consistent between settings.^4^ The reduced liver disease we observed in coinfected individuals highlights the positive impact of wider treatment access, which whilst prescribed to treat HIV, is also inadvertently suppressing HBV. In recent years, there have been increased calls for the integration of hepatitis care into HIV programmes, in order to capitalise on pre-existing infrastructure for screening and treatment.^33^^,^^34^ Previous studies have indicated that <25% of HIV-positive individuals are likely to be screened for HBsAg,^35^ indicating that integration of the screening programmes may be of benefit to both coinfected and monoinfected patients.

We found no difference in the prevalence of elevated hepatic transaminases between monoinfected and coinfected patient groups. It is important to recognise that appropriate thresholds for the ULN of liver enzymes have not been clearly determined for African populations,^21^ and that significant evidence of progressive liver disease can be present even in the absence of abnormalities in liver enzymes. This highlights the lack of sensitivity of liver function tests as biomarkers; clinical surveillance may need to incorporate other markers, laboratory-based fibrosis scores, and/or or elastography results.

### HCC in HBV infection

HCC can arise independent of liver fibrosis,^16^ and therefore even among HBV-infected individuals with no objective evidence of inflammatory or fibrotic liver disease, there is a potential for the emergence of malignancy. In this study, HCC was only documented in three patients so we are unable to draw conclusions that can be generalised, but we note that it arose only in the context of HBV monoinfection. Treatment with nucleoside analogues has been shown to lower the risk of HCC.^36^ Our results suggest that adults with HBV monoinfection may be coming to clinical care too late, and may not have sufficient access to antiviral therapy even when enrolled in clinical care. Further investigation is warranted, but our observations underline a pressing need for simplified, scalable approaches to early HBV diagnosis and treatment.^37^

### HBV suppression on therapy

Despite the immunological defect associated with HIV infection (associated with loss of CD4+ T cells) and the higher prevalence of HBeAg+ status in this group, HBV viraemia can be successfully suppressed in the majority of coinfected patients using conventional TDF-based cART regimens. However, even on appropriate antiviral therapy, over a quarter of patients still had detectable HBV viraemia. TDF therapy can be slow to suppress HBV DNA to below the limits of detection,^38^ but given the median exposure of 15 months, the HBV viraemia we observed on therapy is more likely to be explained by suboptimal adherence to therapy, pharmacokinetics (drug absorption, levels within liver tissue), or drug resistance. Conclusions about adherence must be made with caution, as HIV rebound takes much longer than HBV in patients on efavirenz (EFV), due to the long half-life of this agent, that may be extended still further in patients with CYP2B6 mutations.

Even on treatment, HBV is more likely to be suppressed in the context of HIV co-infection, perhaps related to better support and education around treatment adherence, or potentially to therapy with more than one HBV active agent (TDF+3TC) in the co-infected group. Careful clinical surveillance, with checking and reinforcement of therapy, is therefore important. There is also a need to consider a potential role for dual HBV therapy, and to evaluate the currently uncertain impact of TDF resistance.^8^^,^^39^

### Changes to cART regimens with influence on HBV therapy

Changes to cART recommendations are developing, based on the success of dolutegravir (DTG), both in the context of prior viraemic suppression^40^ and for salvage after failure of first-line treatment.^41^^,^^42^ DTG/3TC is a common combination, but other regimens do not contain any first-line HBV active agents, (e.g. DTG + rilpivirine (RPV),^43^ emtricitabine (FTC),^44^ or ritonavir-boosted lopinavir (LPVr)^42^). These regimens are deemed inappropriate in the context of HBV coinfection,^45^ although there are data demonstrating a potential role for FTC together with TDF in mediating HBV suppression.^46^ As dual therapy combinations enter clinical practice for HIV treatment in Africa, enhanced awareness and scrutiny will be critical to ensure that HBV-positive individuals are diagnosed and receive appropriate treatment.

### Drug resistance

We identified a patient with phenotypic evidence of 3TC-resistant HBV, consistent with the well-documented selection of resistance by 3TC monotherapy,^47^ and in keeping with another African study that documented a high prevalence of 3TC resistance among coinfected patients^4^. TDF resistance is less well substantiated, and can be difficult to confirm phenotypically due to the slow rate of HBV DNA suppression after institution of therapy; however, there are reports confirming the potential for resistance to this agent.^8^^,^^39^

### Caveats and limitations

We have studied a small cohort, representing a distinct geographical setting and focusing on clinical care delivery within the specific environment of a tertiary care university hospital. The study design was observational, incorporating the influence of bias in referral patterns and clinical follow-up, and capturing the influence of systematic differences in guidelines for HBV and HIV management. Based on this cross-sectional view, long-term outcomes of liver disease can only be extrapolated with caution. We have not been able to capture data regarding important influences on HBV and HIV viral loads, including compliance with treatment and the presence of antiviral resistance. HBV genotype data were not available, but may add future insights.^7^^,^^48^ In the longer term, it would be desirable to undertake prospective studies in larger cohorts, based on more robust, reproducible measures of liver disease that have been well validated for the population in question.

Even in the context of an urban teaching hospital, missing data are problematic. Assessment with elastography is not consistently deployed, and serological markers are expensive, and not routinely undertaken at each clinic visit. The difference in fibroscan rates between monoinfected and coinfected patients most likely relates to the combined influence of differences in staffing, resources and equipment. It is also possible that this practice is influenced by a greater perceived risk of developing inflammatory liver conditions in coinfected patients.^49^ Amongst the coinfected group, who were more likely to have undergone assessment, 10% of individuals were nevertheless still lacking elastography results. We were unable to determine specific treatment duration for a proportion of the patients studied, adding to uncertainty about exposure to antiviral therapy in HBV and HBV/HIV infected groups. Our data exemplify a ‘real world’ cohort and highlight genuine complex day-to-day clinical challenges in a LMIC setting.

We used platelet count as a marker of liver disease, but this is a non-specific approach. The sensitivity and specificity of APRI and FIB-4 varies between settings, with recent studies suggesting that cut-off thresholds for patients with chronic HBV may need revising.^50^ Further evaluation is specifically required in African populations,^21^ particularly in the context of high HIV prevalence.^51^ The gamma-glutamyl transpeptidase to platelet ratio (GPR) may be a more accurate biomarker of liver disease,^21^^,^^52^ although poor correlation with elastography scores is reported in HIV coinfected individuals.^51^ In this study, we were unable to determine GPR as the extended panel of liver function tests required is not routinely collected.

Our observations are inevitably influenced by referral bias: patients with more advanced pathology are most likely to be diagnosed and referred for tertiary-level assessment, and more likely to remain under long term hospital follow-up. In the general population, the majority of cases of HBV monoinfection are undiagnosed, and even those with a diagnosis are infrequently under routine surveillance, less likely to be stratified for treatment, and rarely receive a regular supply of appropriate therapy supported by clinical monitoring.^18^

### Conclusions

Our results demonstrate the potential impact of differences between referral, surveillance and therapy for HBV monoinfection in contrast to HIV/HBV coinfection. By describing a neglected burden of liver disease associated with CHB in this setting, we point to a need for a reappraisal of guidelines, and enhanced investment of resource, clinical care and infrastructure to provide a safer and more equitable service for patients with HBV monoinfection. High standards of provision of HIV services, with consistent access to diagnosis, surveillance and TDF-based treatment, can be advantageous in delivering a high standard of clinical care for those with HBV coinfection. In order to make progress towards 2030 elimination goals, service providers should capitalise on the existing infrastructure and investment deployed for HIV as both a precedent and a foundation for improved clinical care for HBV infection.

## Declaration of Competing Interest

Nil to declare

## Appendix Supplementary materials

Image, application 1
