# Supplementary material for: Treatment advantage in HBV/HIV coinfection compared to HBV monoinfection in a South African cohort
Source: J Infect. 2020 Jul;81(1):121–30. doi: 10.1016/j.jinf.2020.04.037 (PMC7308798; doi:10.1016/j.jinf.2020.04.037)
Supplement: Supplementary file 1 [file mmc1.zip › 191212 Suppl Table 1 STROBE Statement.pdf]

Treatment advantage in HBV/HIV coinfection compared to HBV monoinfection in a South African cohort

Maponga *et al*.

Suppl table 1: STROBE Statement

Requirement for manuscript	Inclusion in manuscript
**Title and abstract**	
Indicate the study's design with a commonly used term in the title or the abstract	Cross-sectional observational cohort study described by abstract.
Provide in the abstract an informative and balanced summary of what was done and what was found	This is provided in the structured abstract.
**Introduction**	
Explain the scientific background and rationale for the investigation being reported	This information is covered by the introduction, with supporting references.
State specific objectives, including any prespecified hypotheses	Aims of the study are set out in the abstract and introduction, including hypotheses on which our work was based
**Methods**	
Present key elements of study design early in the paper	This is a cross-sectional observational cohort study, recruited through prospective approach over a year; the approach is described by the abstract, introduction and methods sections.
Describe the setting, locations, and relevant dates, including periods of recruitment, exposure, follow-up, and data collection	These details are all included in the methods section.
Cohort study—Give the eligibility criteria, and the sources and methods of selection of participants. Describe methods of follow-up	Eligibility and recruitment described in methods. Follow-up not applicable.
Cross-sectional study—Give the eligibility criteria, and the sources and methods of selection of participants	Eligibility and recruitment described in methods.
Clearly define all outcomes, exposures, predictors, potential confounders, and effect modifiers. Give diagnostic criteria, if applicable	Diagnostic criteria for HBV infection are described in methods. Confounders are addressed in discussion.
**Data sources and analysis**	
For each variable of interest, give sources of data and details of methods of assessment (measurement). Describe comparability of assessment methods if there is more than one group.	Sources of clinical, laboratory, imaging data are described in methods. Assessment methods are the same in both groups (HIV positive vs HIV negative).

Describe any efforts to address potential sources of bias	Bias is introduced by the recruitment setting of the study, which we could not change. However, we have explained the nature and potential impact of this bias in the discussion section.
Explain how the study size was arrived at	The study was a planned interim analysis after one year of recruitment – this is included in methods.
Explain how quantitative variables were handled in the analyses. If applicable, describe which groupings were chosen and why	Handling of data are described in methods. Rationale for comparing HIV positive vs HIV negative is set out in introduction.
Describe all statistical methods, including those used to control for confounding	Our methods are all included in the statistical analysis section.
Describe any methods used to examine subgroups and interactions	No subgroup analysis was undertaken, due to small sample size.
Explain how missing data were addressed	The denominator is corrected to account for missing data. Table 1 legend contains detailed information about number of participants for whom each parameter was available.
Cross-sectional study—If applicable, describe analytical methods taking account of sampling strategy	Not applicable – participants were recruited consecutively and prospectively (no specific sampling strategy applied).
Describe any sensitivity analyses	Not applicable.
**Results**	
Report numbers of individuals at each stage of study—eg numbers potentially eligible, examined for eligibility, confirmed eligible, included in the study, completing follow-up, and analysed	This is shown in flow diagram (figure 1)
Give reasons for non-participation at each stage	Not applicable (no intervention, no longitudinal follow up, so no drop outs following recruitment).
**Participants**	
Consider use of a flow diagram	Included in paper as figure 1.
Give characteristics of study participants (eg demographic, clinical, social) and information on exposures and potential confounders	This information is included in table 1.
Indicate number of participants with missing data for each variable of interest	Missing data are summarised based on the denominators in Table 1 (full metadata also published alongside the paper).

**Descriptive data**	
Cohort study—Summarise follow-up time (eg, average and total amount)	Not applicable - no longitidunal follow up was undertaken.
Cohort study—Report numbers of outcome events or summary measures over time	Oucome events (chronic liver disease) are reported in Table 1 and results section.
**Outcome data**	
Cross-sectional study—Report numbers of outcome events or summary measures	Outcomes are recorded on a cross-sectional basis, and the data (absolute numbers as well as proportions) are presented in Table 1 and in an extended suppl data file.
Give unadjusted estimates and, if applicable, confounder-adjusted estimates and their precision (eg, 95% confidence interval). Make clear which confounders were adjusted for and why they were included	For this small dataset, confidence intervals would be very wide and not helpful to contribute to data interpretation. We highlight potential confounders in the discussion, but did not adjust for these, again based on small sample size.
Report category boundaries when continuous variables were categorized	Boundaries are stated in the methods (with references), presented in Table 1 footnores, and indicated on figures.
**Main results**	
If relevant, consider translating estimates of relative risk into absolute risk for a meaningful time period	Not applicable (Number of participants in study too small and no longitudnal analysis undertaken).
Report other analyses done—eg analyses of subgroups and interactions, and sensitivity analyses	Not applicable.
**Discussion**	
Summarise key results with reference to study objectives	This is included in the first section of the discussion.
Discuss limitations of the study, taking into account sources of potential bias or imprecision.	Section entitled 'caveats and limitations' is included in the discussion, including sources of bias.
Discuss both direction and magnitude of any potential bias	As above, this is included in the discussion.
Give a cautious overall interpretation of results considering objectives, limitations, multiplicity of analyses, results from similar studies, and other relevant evidence	Caveats and limitations are clearly stated in the discussion. We present our results alongside references to other comparable studies to compare and contrast.
Generalisability: Discuss the generalisability (external validity) of the study results	We have explained why caution is needed in extrapolating results, while setting out the utility of our findings for the wider community.
**Other information**	
Give the source of funding and the role of the funders for the present study and, if applicable, for the original study on which the present article is based	Funding information is presented at the end of the article (Wellcome grant ref 110110).
