# Supplementary material for: Treatment advantage in HBV/HIV coinfection compared to HBV monoinfection in a South African cohort
Source: J Infect. 2020 Jul;81(1):121–30. doi: 10.1016/j.jinf.2020.04.037 (PMC7308798; doi:10.1016/j.jinf.2020.04.037)

SUPPLEMENTARY FIGURES

Treatment advantage in HIV/HBV coinfection compared to HBV monoinfection in a South African cohort

Tongai G Maponga et al.

Suppl Fig 1: Ethnic origins (birth country) of participants enrolled into a cross-sectional cohort of HBV infection in Cape Town, South Africa.

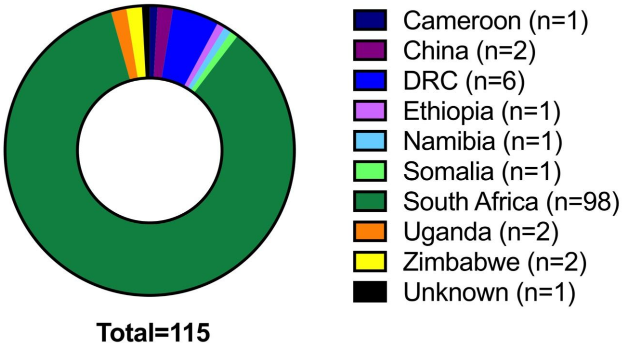

Suppl Fig 2: Plasma viral loads for HBV and HIV in a cross-sectional study of adults in Cape Town, South Africa. (A) Distribution of HBV DNA viral load in individuals with HBV monoinfection and HBV/HIV coinfection; horizontal lines indicate median with whiskers representing IQR; the numbers shown in brackets under each category report the number of individuals represented; p-value by Mann Whitney U test. (B) Relationship between HIV viral load and HBV viral load in serum, with R^2 and p values by linear regression based on $\log_{10}$ viral load; note the point where HIV and HBV VL=0 represents 15 individuals.

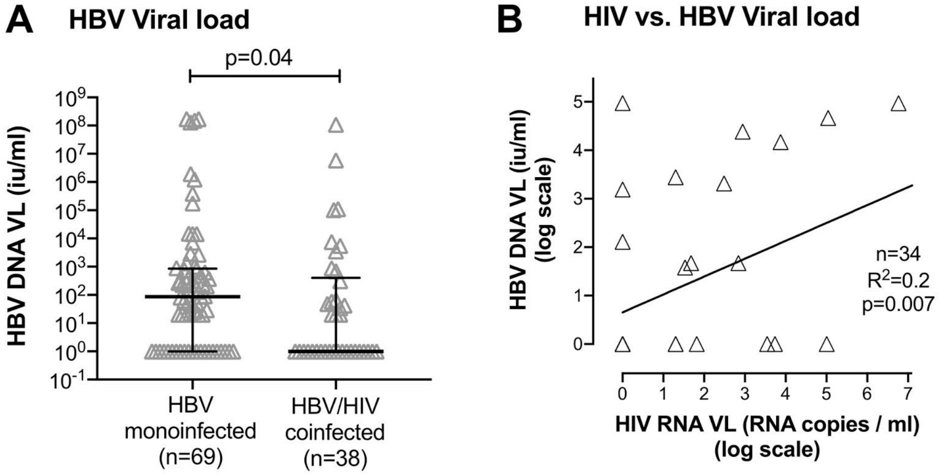

Supplement: Supplementary file 1 [file mmc1.zip › 191212 Suppl Figures 1 and 2.pdf]
